# Gender Differences in Subacute Post-Stroke Patients During Rehabilitation: Functional, Cognitive, and Nutritional Insights

**DOI:** 10.3390/neurolint17120193

**Published:** 2025-11-30

**Authors:** Carola Cocco, Mariacristina Siotto, Alessandro Guerrini, Marco Germanotta, Francesca Falchini, Valeria Cipollini, Laura Cortellini, Arianna Pavan, Stefania Lattanzi, Sabina Insalaco, Dionysia Papadopoulou, Elisabetta Ruco, Erika Antonacci, Irene Giovanna Aprile

**Affiliations:** 1 IRCCS Fondazione Don Carlo Gnocchi ONLUS, 50143 Florence, Italy; ccocco@dongnocchi.it (C.C.); aguerrini@dongnocchi.it (A.G.); mgermanotta@dongnocchi.it (M.G.); ffalchini@dongnocchi.it (F.F.); vcipollini@dongnocchi.it (V.C.); lcortellini@dongnocchi.it (L.C.); apavan@dongnocchi.it (A.P.); slattanzi@dongnocchi.it (S.L.); sinsalaco@dongnocchi.it (S.I.); dpapadopoulou@dongnocchi.it (D.P.); eruco@dongnocchi.it (E.R.); eantonacci@dongnocchi.it (E.A.); iaprile@dongnocchi.it (I.G.A.); 2 Department of Science and Technology for Humans and the Environment, Università Campus Bio-Medico di Roma, 00128 Rome, Italy

**Keywords:** post-stroke, gender, rehabilitation, activity of daily living, cognitive impairment, nutritional status, malnutrition, oxidative stress, food consumption

## Abstract

**Background/Objectives:** Despite the well-documented gender differences observed during hospitalisation, research in post-stroke recovery remains limited. This study aims to clarify this topic in subacute post-stroke patients undergoing rehabilitation, considering not only functional and cognitive outcomes but also nutritional status and food consumption. **Methods**: At admission (T0), patients were assessed for demographic, anamnestic, and clinical data and were diagnosed for malnutrition according to the Global Leadership Initiative on Malnutrition (GLIM) criteria. At T0 and after a six-week rehabilitation program (T1), nutritional status was assessed by anthropometric measurements, serum analysis of albumin, glucose, lipidic, metal, and oxidative stress panel, and the calculation of the Geriatric Nutritional Risk Index; food consumption was recorded daily. Functional independence in Activities of Daily Living was measured at both T0 and T1 by the modified Barthel Index (mBI), and cognitive impairment was assessed by the Montreal Cognitive Assessment (MoCA), adjusted for age and education. **Results**: We enrolled 87 patients (mean age 69 ± 12 years; 42 women and 45 men); of these 52.4% of women were malnourished, compared to 33.3% of men. After rehabilitation (T1), women showed higher oxidative stress (549 ± 143 vs. 491 ± 121 UCARR; *p* = 0.041) and poorer functional outcomes (55.3 ± 26.1 vs. 67.1 ± 21.8; *p* = 0.032), despite similar cognitive improvements (19.5 ± 6.4 vs. 21.9 ± 5.2; *p* = 0.060) compared with men. **Conclusions**: This study highlights the importance of personalised treatment strategies that account for gender-specific differences to optimise recovery in post-stroke patients.

## 1. Introduction

Stroke is the leading cause of adult disability [1,2] and the second leading cause of death worldwide, imposing a significant burden on patients, their families, and healthcare systems. The incidence of stroke is expected to increase as the number of people over the age of 65 increases, posing significant challenges for clinicians and policy makers in the near future [3].

Each year, 53% of all strokes occur in women, and globally, women account for just over half (56%) of all people who have experienced stroke. It is known that both the incidence and prevalence of ischaemic stroke are higher in women (55% and 57%, respectively) [3]. Women have a higher risk of stroke and this may be related to longer life expectancy as well as the influence of specific hormonal factors [4,5]. They have a stronger correlation with obesity, a higher prevalence of atrial fibrillation, and a higher risk of hypertension even after antihypertensive treatment. Furthermore, compared to men, women have worse outcomes in terms of function and quality of life after a stroke, due to their older age at stroke onset, higher stroke severity, and higher prevalence of risk factors for cognitive impairment [6].

To date, despite much knowledge about the various aspects of stroke, there are still few studies that have investigated the differences in functional and cognitive recovery between men and women after a stroke.

Some studies indicated better recovery rates in men in terms of the activities of daily living (ADL) [7,8,9]; one interesting study showed that when each item of the functional independence measure was considered separately, women performed as well or better than men on most items [10]. Additional studies showed no differences between genders in recovery [11,12]. In contrast, in another study, older women showed better functional recovery than men of the same age [13].

To date, few studies have examined gender differences in the cognitive recovery of post-stroke patients, with variable results. One study found that women had worse cognitive outcomes than men 90 days after stroke. These differences were attributed to sociodemographic factors and a worse pre-stroke condition [6]. However, the results of another study [14] indicated that men and women have similar overall cognitive results after a stroke, when demographic and lesional factors are considered. Although men and women differed in their performance on some individual cognitive tests, no significant disparities were identified in the overall performance levels between the genders [14].

Adequate nutritional support during rehabilitation can enhance brain neuroplasticity processes, promote neuroprotection, and improve muscle strength, contributing to better functional outcomes, cognitive performances, and overall quality of life [15]. Nutritional impairment is a common risk for stroke survivors, and malnutrition has been identified as a recurrent complication, especially in elderly patients, correlating with poor independence in the activities of daily living and poor quality of life [16,17,18]. Malnutrition is frequently undiagnosed and untreated not only during hospitalisation for acute treatment [19] but also throughout or after rehabilitation following a stroke [20]. Despite the significant frequency of malnutrition among post-stroke patients, very few studies have analysed the impact of the nutritional status on rehabilitation outcomes [15,16,21,22,23,24], with women seeming to be more susceptible to malnutrition [25,26,27]. This could depend on several factors, both physiological and psychological, including a higher tendency to have strokes later in life and post-stroke depression [28].

Another aspect that has been demonstrated to be strongly linked to nutritional status and stroke pathophysiology is the systemic oxidative stress (OS). An alteration in OS has been linked to a reduction in brain plasticity, long-term synaptic potentiation, and synaptic signalling, showing an involvement in the recovery of neurological deficits and neurorehabilitation [24,29]. Very high levels of OS have been found in post-stroke patients in the acute phase [30,31]. Our group demonstrated that in the subacute phase, the very high levels observed at admission tended to diminish after the rehabilitation period and were negatively correlated with rehabilitation outcomes [32]. Furthermore, a previous study by our group revealed that women had lower antioxidant reserves [23]. These findings suggest a compelling relationship between nutrition, oxidative damage, and post-stroke recovery. In fact, the correct intake of exogenous antioxidants in the diet could support the activity of endogenous antioxidants and help mitigate the damaging effects of the reactive oxygen and nitrogen species cascade after stroke insult, preventing further neural damage and ultimately promoting recovery.

To our knowledge, few studies have explored gender differences in subacute stroke patients undergoing rehabilitation, analysing in detail and globally the nutritional status together with the rehabilitation outcomes. Therefore, the aim of this study is to describe the differences between women and men in a cohort of subacute stroke patients in terms of nutritional status including systemic oxidative stress status, food consumption during the hospitalisation period, and rehabilitation outcomes.

## 2. Materials and Methods

### 2.1. Study Design and Participants

This monocentric observational study analysed a group of subacute post-stroke patients recruited to the centre of Fondazione Don Carlo Gnocchi “S. Maria della Provvidenza” in Rome (RM, Italy) between September 2020 and April 2023. This study pertains to a multicentric protocol registered on ClinicalTrials.gov (short title: NUTRISTROKE; identification number: NCT04923165, registered on 4 September 2020). The study was conducted in accordance with the Declaration of Helsinki and approved by the Ethics Committee of Fondazione Don Carlo Gnocchi, Milan on 12 February 2020, with a non-substantial amendment on 14 October 2020 (Prot.n.22/2020/CE_FdG/FC/SA_14/10/20). Informed consent was obtained from all subjects involved in the study, after a detailed explanation of the study’s aims and rehabilitation protocols.

Patients with their first stroke were enrolled and evaluated at admission (T0) and after a 6-week rehabilitation program (T1).

The inclusion criteria were as follows: (i) first ischaemic or haemorrhagic stroke, confirmed by magnetic resonance imaging (MRI) or computed tomography (CT); (ii) age between 18 and 85; (iii) less than six months since the stroke insult; (iv) adequate cognitive and language abilities to comprehend the instructions for administering the assessment scales and to sign the informed consent.

Patients were excluded following these criteria: (i) a previous stroke; (ii) behavioural and cognitive disorders and/or reduced compliance, which would hinder active therapy or the understanding and signing of informed consent. Moreover, at enrolment and during evaluation, patients were not infected with SARS-CoV-2. This observational study was conducted in accordance with the STROBE 2019 guidelines. The completed STROBE checklist is provided in Supplementary Table S1.

### 2.2. Rehabilitation Treatment

Patients completed a rehabilitation regimen that included traditional physical therapy, which was carried out for 45 min each day, six days a week. The rehabilitation treatment consisted of passive, active-assisted, and active mobilisations, exercises for muscle strength recovery, stretching, functional and task-oriented training, proprioceptive exercises, postural passages and transfers, sitting and standing training, motor coordination and balance training, walking training, and activities of daily living recovery training. Additionally, utilising a set of robotic devices, each patient underwent robotic treatment of the upper limb five times each week for 45 min at a time. The following robotic devices were employed: Motore (Humanware Srl, Pisa, Italy), Amadeo (Tyromotion, Graz, Austria), Diego (Tyromotion, Graz, Austria), and Pablo (Tyromotion GmBH, Graz, Austria), as detailed in previous studies [33,34]. Patients through upper limb robotic therapy conducted both motor and cognitive tasks, with the aid of visual and audible input from the equipment.

### 2.3. Clinical, Activity of Daily Living, and Cognitive Impairment Assessment

The demographic, anamnestic, and clinical data at admission (T0) were recorded. The disease burden was measured using the 56-point Cumulative Illness Rating Scale (CIRS) [35].

The Modified Barthel Index (mBI) was employed both at T0 and T1, which is an ordinal scale used to assess independence in ADL ranging from 0 to 100, with lower scores denoting increased disability [36]. The mBI contains 10 items, with each designed to assess a specific aspect of patient’s ability to perform different activities (feeding, personal hygiene, dressing, bathing, bladder control, bowel control, toilet transfers, stair climbing, and ambulation/wheelchair).

Cognitive impairment was assessed at T0 and at T1 using the Italian version of the Montreal Cognitive Assessment (MoCA). The MoCA is composed of various subtests to assess abstraction, set shifting, and cognitive flexibility. The total MoCA score ranges from 0 to 30, with higher scores reflecting superior overall cognitive performance. The final MoCA value was calculated using adjustment grids according to age and education, both for the total MoCA values and for subtest raw scores [37].

### 2.4. Nutritional Status Assessment

The nutritional status assessment was performed by means of anthropometric measurements, screening and diagnosis of malnutrition by GLIM criteria, and haematochemical assessment, in terms of albumin, glucose, lipid panel, metal panel, and oxidative stress panel. Additionally, the Geriatric Nutritional Risk index was calculated.

#### 2.4.1. Anthropometric Measurements

Height was measured at T0 for each patient who could stand, with data reported in metres (m). The height of participants unable to stand was measured considering the knee height, using the Chumlea equation [38]. Body weight was measured at both T0 and T1. Patients who could stand were weighed on a calibrated scale (Seca 750, Seca, Hamburg, Germany), whereas those who could not stand were weighed on a chair scale (Wunder DE5, Wunder Sa.Bi. srl; Milan, Italy). Weights were recorded in kilogrammes (kg), with accuracy to the closest 0.1 kg. The body mass index (BMI, kg/m^2^) at T0 and T1 was then determined.

#### 2.4.2. Malnutrition Screening and Diagnosis

Patients were screened at T0 for malnutrition risk with the Mini Nutritional Assessment Short-form (MNA-SF^®^), a simple, quick, and easy nutritional tool included in the full Mini Nutritional Assessment (MNA^®^) [39]. The MNA-SF^®^ includes six assessment questions about nutritional and health condition. Patients were considered at “normal nutritional status” if MNA-SF^®^ screening score values were from 12 to 14, at “risk of malnutrition” with values between 8 and 11, and “malnourished” with a score ≤ 7. For scores ≤ 11, the diagnosis of malnutrition was made based on the presence of at least one phenotypic and at least one etiological criterion, according to GLIM criteria [40] and as detailed in a previous study [21].

#### 2.4.3. Haematochemical Analyses

The blood samples of patients were collected in the early morning (7:30–9:00 a.m.) after an overnight fast to standardise the assessment of those biochemical variables that are affected by the circadian cycle and food intake. Sera samples were separated through centrifugation (3000 rpm, 10 min, and 4 °C), divided into 0.5 mL aliquots, and rapidly stored at −80 °C. Subjects’ and reference samples were thawed just before the assays. All the analyses were performed in duplicate, both at T0 and at T1, and tested on an integrated analytical photometer (Free Carpe Diem, Diacron International SRL, Grosseto, Italy).

Albumin levels were measured by bromocresol colorimetric assay [41]; glucose was assessed by an oxidase/peroxidase system [42]. Total cholesterol was measured by means of oxidation from a cholesteroxidase to cholest-4-en-3-one [43] while direct HDL cholesterol was assessed by a transformation of the HDL portion into a quinone derivative [44]; triglycerides were measured by a peroxidase-coupled method [45]. Calcium was analysed using a method based on the o-cresolphthalein complexone test [46], while magnesium was assessed using a direct colorimetric assay based on the xylidyl Blue-I method [47]. Serum iron levels were measured by a colorimetric assay based on Ferene S. [48].

The oxidative stress panel was analysed as detailed in Cocco et al. [32]. In particular, the colorimetric determination of hydroperoxides content was assessed by the dROMs test. The global antioxidant defences, as for endogenous and exogenous molecules present in plasma, was measured by BAP test.

The oxidative stress index (OSI) was calculated as the ratio of BAP to d-ROMs, which normalises the relative antioxidant capacity to the total circulating hydroperoxides. This antioxidative/oxidative balance reflects the body’s potential antioxidant capacity, as described in the literature [49,50]. A threshold value of 7.3 was used to classify the OSI: values below 7.3 were considered indicative of an oxidised state, while values equal to or above 7.3 were classified as a reduced state [49,50].

The thiol groups of serum biomolecules, which are a significant component of the antioxidant plasma barrier, were tested with SHp test, using the Ellman method [51].

All kits were furnished by Diacron (Diacron International SRL, Grosseto, Italy).

We calculated the Geriatric Nutritional Risk (GNRI) to assess the risk of nutritional-related complication as detailed in a previous work [23].

### 2.5. Food Consumption

The meals were planned and prepared by our rehabilitation centre canteen in accordance with the Italian recommendations “National Recommended Energy and Nutrient Intake Levels for the Italian Population” (LARN) [52]. Based on Italian tradition, the meals served at lunch and dinner included three main dishes called “first dish”, “second dish”, and “side dish”, as well as an additional dish consisting of fruit. The “first dish” is primarily composed of carbohydrates, such as pasta, rice, or semolina, and is usually seasoned with legumes or vegetables, or both. The “second dish” consists mainly of protein sources such as meat, fish, eggs, or dairy products. The side dish consists of seasonal vegetables. Fruit is usually a whole fruit (such as apple, banana or orange) or a mousse. Patients with dysphagia eat the same food but with changes to the consistency or consume liquids.

Patients’ food consumption during the six-week study was assessed by means of the visual “plate waste” estimation method [23,53,54]. Nurses and speech therapists kept track of patients’ meal waste (breakfast, lunch, and dinner) for six weeks, six days a week. Scores ranged from 0 to 4 on a 5-point scale (0 = not wasted, 1 = ¼, 2 = ½, 3 = ¾, or all wasted), as described in a previous study [21]. The average daily dish waste score was then determined for all 108 meals consumed by all patients and also separately for the “first dish”, “second dish”, “side dish”, and “fruit plate”. The data were aggregated into 5 categories: (i) the “totality of all meals”, which includes the total consumption of all meals consumed for six weeks; (ii) the “first dish” (lunch + dinner) consisting of carbohydrates (e.g., cereals such as pasta, rice, or semolina); (iii) the “second dish” (lunch + dinner) consisting of a protein source (meat, fish, eggs, or dairy products); (iv) the “side dish” (lunch + dinner), consisting of a vegetable dish; (v) the “fruit dish”, usually consisting of a mousse or a seasonal fruit, such as an orange, a banana, or an apple. For dysphagic patients, meals were served with the same food modified in consistency or fluid.

### 2.6. Statistical Analysis

Descriptive statistics were used to express the demographic and clinical characteristics of patients, with numerical data expressed as the mean (SD) and categorical data presented as counts and percentages. The sample size was calculated assuming a type I error (α) of 0.05, a power (1 − β) of 80%, and an expected weak-to-moderate correlation between nutritional parameters and stroke recovery (r = 0.30). Based on these assumptions, a total of 85 patients is required, accounting for 5%, we planned to enrol a total of 91 subjects (G-Power 3.1.9.7). Analysis of normality was performed with the Shapiro–Wilk test, and non-parametric tests were employed accordingly. To compare the demographic and clinical characteristics between women and men, as well as the nutritional status variables both at T0 and at T1, the Mann–Whitney U test or the chi-squared test was employed, as appropriate. To evaluate the changes in the ADL and cognitive impairment following rehabilitation treatment, mBI and MoCA data measured at T0 were compared with those at T1 by means of the Wilcoxon Signed Rank Test; results were analysed for the whole group, as well as for men and women separately.

For all the statistical analysis, a *p*-value lower than 0.05 was deemed as significant. Statistical analysis was performed using IBM SPSS Statistics for Windows (Version 28.0. IBM Corp.: Armonk, NY, USA).

## 3. Results

### 3.1. Participants and Baseline Characteristics

We screened 140 patients, and we enrolled 91 subjects at the Fondazione Don Carlo Gnocchi “S. Maria della Provvidenza” Centre in Rome (RM, Italy); among these, 87 completed the study (42 women, 45 men; mean age 69 ± 12 years) All participants were evaluated at baseline (T0) and after a six-week rehabilitation program (T1). A detailed flow chart of the study is available in Supplementary Figure S1.

The baseline characteristics (demographic and clinical features, disability assessment) are reported for the whole group and differentiated for women and men in Table 1. The sample was homogeneous, although the distribution of past heart and thyroid disease differed between the two sexes.

### 3.2. Nutritional Status

As shown in Table 2, the nutritional status of patients at admission (T0) is reported for the whole group and disaggregated by gender. The MNA-SF^®^ screening identified 83 subjects at risk of malnutrition (95.8%, 43 women and 40 men). Following the GLIM criteria, 37 individuals (42.5%) were malnourished. Specifically, malnutrition affected 22 out of 42 women (52.4%) and 15 out of 45 men (33.3%).

As expected, the anthropometric measurements were different between women and men, while no significant differences were observed in haematochemical parameters, except for HDL cholesterol, which was higher in women but below the normal cut-off (>65 mg/dL in women and > 55 mg/dL in men) in both genders.

Both women and men had very high values of hydroperoxides (dROMs) (>500 UCARR) and lower levels of OSI (<7.3) with respect to normal values. Women tended to have slightly higher values of dROMs and lower values of total antioxidants (BAP), without any statistical significance.

Table 3 shows the nutritional status after six weeks of rehabilitation treatment (T1) in the whole group and disaggregated by gender. The weight measurements remained, as expected, significantly different.

At T1, no significant differences were observed between the two genders in haematochemical parameters, except for total and HDL cholesterol, which were both higher in women. In both men and women, the total cholesterol values were within the normal range (<200 mg/dL), while the HDL-cholesterol was below the gender-specific normal values (>65 mg/dL in women and >55 mg/dL in men). Regarding the oxidative stress panel, women had higher levels of hydroperoxides than men (549 ± 143 vs. 491 ± 121 UCARR; *p* = 0.041). However, both women and men still showed very high levels of oxidative stress and lower levels of OSI than the normal reference, demonstrating an imbalanced systemic antioxidant capacity.

### 3.3. Food Consumption During the Rehabilitation Period

**Figure 1:**
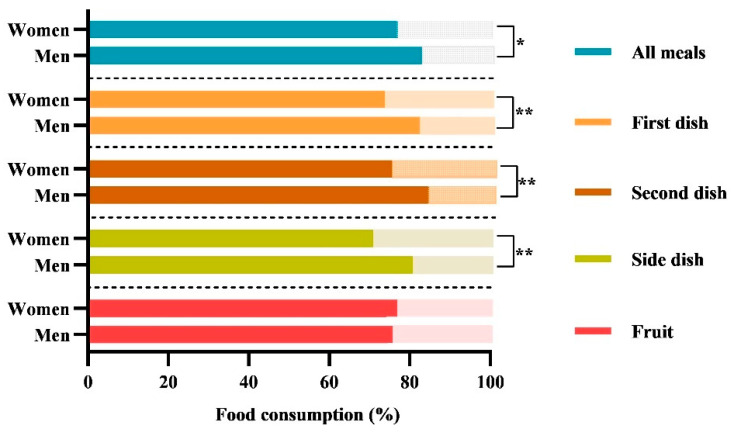
**Comparison of food consumption between women and men.** The histograms show the percentages of the daily average food consumption of women and men during the six weeks of treatment. The following nomenclature is employed for the consumption of meals served in accordance with Italian customs: (i) the totality of “all meals”; (ii) the “first dish” (lunch + dinner) consists of carbohydrates (e.g., cereals such as pasta, rice, or semolina), which are typically seasoned with vegetables or legumes or both; (iii) the “second dish” (lunch + dinner) consists of a protein source (meat, fish, eggs, or dairy products); (iv) the ‘side dish’ (lunch + dinner), consists of a vegetable dish; (v) the “fruit”, consists of mousse or a seasonal fruit, such as an orange, a banana, or an apple. The data are reported as the mean percentage (%). The *p*-value refers to the Mann–Whitney test; * *p*-value < 0.05; ** *p*-value < 0.01.

As shown in Figure 1, the food consumption during the six weeks of observation was different between women and men. Women discarded more food than men, specifically in terms of the “totality of all meals” (23% vs. 17%; *p* = 0.012), “first dish” (26% vs. 17%; *p* = 0.012), “second dish” (24% vs. 15%; *p* = 0.003), and “side dish” (29% vs. 19%; *p* = 0.004), while we did not observe differences for the fruit.

**Figure 2:**
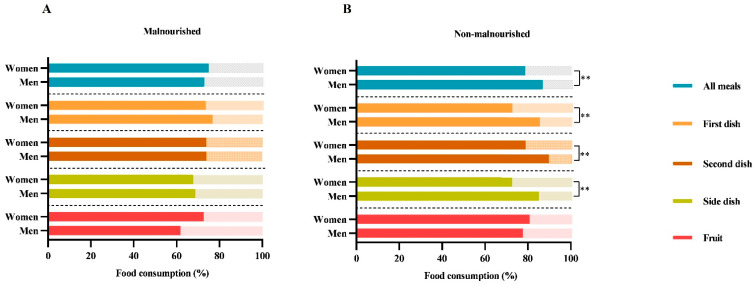
**Food consumption differences between women and men in malnourished** (**A**) **and non-malnourished patients** (**B**). The histograms show the differences in food consumption of meals served according to Italian customs between the two genders by dividing the subjects by malnutrition (“malnourished” and “non-malnourished”), according to GLIM criteria. The following nomenclature is employed for the consumption of meals served in accordance with Italian customs: (i) the totality of “all meals”; (ii) the “first dish” (lunch + dinner) consists of carbohydrates (e.g., cereals such as pasta, rice, or semolina), which are typically seasoned with vegetables or legumes or both; (iii) the “second dish” (lunch + dinner) consists of a protein source (meat, fish, eggs, or dairy products); (iv) the ‘side dish’ (lunch + dinner) consists of a vegetable dish; (v) the “fruit” consists of mousse or a seasonal fruit, such as an orange, a banana, or an apple. The data are reported as the mean percentage (%). The *p*-value refers to the Mann–Whitney test; ** *p*-value < 0.01.

Furthermore, as shown in Figure 2, dividing patients by malnutrition, no differences between women and men were observed. On the contrary, in non-malnourished subjects (n = 50), women discarded more food than men, in particular 21% of the “totality of all meals” (21% vs. 13%; *p* = 0.006), 33% of the “first dish” (27% vs. 14%; *p* = 0.004), 21% of the “second dish” (21% vs. 10%; *p* = 0.001), and 27% of the “side dish” (27% vs. 14%; *p* = 0.006).

### 3.4. Activities of Daily Living and Cognitive Impairment

After six weeks of rehabilitation (T1) the whole sample had a significant improvement in the ADL measured with mBI values (45.3 ± 19.5 vs. 61.3 ± 24.6; *p* < 0.001) and cognitive impairment in terms of MoCA values (19.0 ± 6.1 vs. 20.8 ± 5.9; *p* < 0.001).

**Figure 3:**
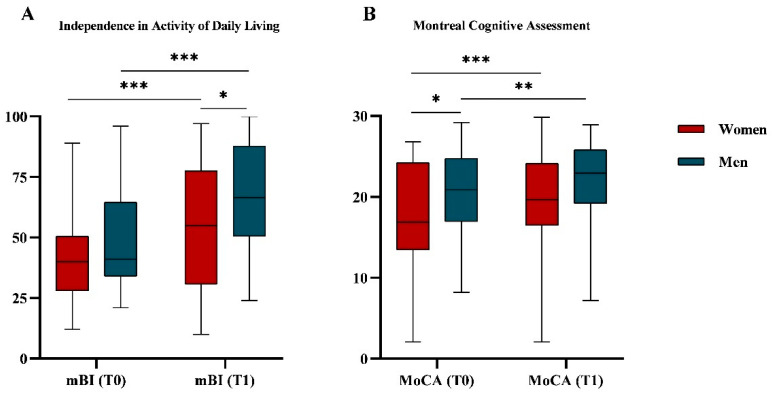
**Functional independence** (**A**) **and cognitive impairment** (**B**) **assessment.** (**A**) Boxplot representing distribution of functional independence assessed by modified Barthel Index (mBI) both at T0 and at T1 and divided by gender. (**B**) Boxplot representing distribution of the cognitive impairment measured with Montreal Cognitive Assessment (MoCA) values both at T0 and at T1 and divided by gender. MoCA scores were adjusted for age and education. The *p*-values refer to the Mann–Whitney test for comparison between genders and the Wilcoxon signed rank test for comparison between T0 and T1. * *p*-value < 0.05; ** *p*-value < 0.01; *** *p*-value < 0.001.

Figure 3 shows the mBI (A) and in MoCA (B) values at T0 and T1, disaggregated by gender. Women and men showed a significant improvement in mBI (41.2 ± 17.3 vs. 55.3 ± 26.1; *p* < 0.001 and 49.5 ± 20.8 vs. 67.1 ± 21.8; *p* < 0.001, respectively) and MoCA values (20.6 ± 5.1 vs. 22.0 ± 5.2; *p* = 0.001 and 17.4 ± 6.7 vs. 19.5 ± 6.4; *p* < 0.001, respectively). Comparing women and men at T0, the MoCA values were different (17.4 ± 6.7 vs. 20.6 ± 5.1; *p* = 0.024), while the mBI values were not (41.2 ± 17.3 vs. 49.2 ± 20.8; *p* = 0.144). On the other hand, at T1, women showed significantly lower mBI values than men (55.3 ± 26.1 vs. 67.1 ± 21.8; *p* = 0.032), while the MoCa values were similar (19.5 ± 6.4 vs. 21.9 ± 5.2; *p* = 0.060).

## 4. Discussion

Although the importance of gender analysis in healthcare is well recognised, few studies have investigated the gender-related differences during rehabilitation treatment after a stroke [9,11,13,55,56,57,58].

In our cohort, the analysis revealed that, at admission, subacute post-stroke women exhibited clinical conditions and nutritional status comparable to those of men, along with a similar level of functional independence in ADL but exhibited higher cognitive impairment. Conversely, during hospitalisation for rehabilitation, they discarded more food. At the end of the six-week rehabilitation period, they showed higher hydroperoxides levels and achieved lower functional independence in ADL, while their cognitive impairment became comparable to that of men. It is important to underline that the sample was homogeneous, and no gender differences in age, latency, level of education, disability, comorbidity, or pharmaceutical treatment were observed.

As reported in the literature [27,59,60] and as discussed in our previous study [21], the majority (95.4%) of subjects admitted to our rehabilitation unit were at risk of malnutrition. The presence of a chronic disease such as stroke, as well as inflammatory processes, feeding difficulties, and functional disabilities is, in fact, associated with an increased risk of malnutrition, especially in elderly patients [25]. Given the advanced age of the subjects (mean 69 ± 12 years), the risk of malnutrition was also influenced by the numerous age-related changes, including reduced physical activity, low appetite, a bedridden state, and neurological deficits affecting oral nutrition [27,60,61]. Moreover, the study found that 42.5% of patients were malnourished, with a higher prevalence among women (52.4%) compared to men (33.3%).

The number of studies assessing gender differences in relation to malnutrition in post-stroke patients remains limited; however, women appear to be more vulnerable to malnutrition, both on admission and after discharge [26,27]. In particular, this may be related to a number of factors, such as a longer life expectancy compared to men or the higher likelihood of experiencing unfavourable economic and social situations in later life [27,62]. Moreover, women are more likely to suffer from more severe strokes, resulting in worse functional status and lower quality of life compared to men, which in turn contributes to a higher susceptibility to malnutrition [26,27].

Despite its significant impact on various clinical outcomes in hospitalised patients, affecting up to 90% of the elderly population, malnutrition remains commonly misdiagnosed and undertreated in hospitals [19]. To prevent the further deterioration of patients’ general condition, it is essential to address malnutrition during prolonged rehabilitation periods. For this reason, a proper nutritional status assessment is necessary to monitor patients. In the current cohort, the analysis of nutritional status revealed normal anthropometric and biological differences between genders. As expected, men had a higher weight and BMI, while women had higher levels of total cholesterol and HDL-cholesterol. In both genders, total cholesterol values were within the normal range (<200 mg/dL). However, HDL-cholesterol levels were below the gender-specific reference values (>65 mg/dL for women and >55 mg/dL for men), which may be related to advanced age and low physical activity levels. These findings are consistent with well-established gender differences and age-related physiological changes. In particular, ageing is associated with a decline in HDL-cholesterol levels, more evident in men, and with an increase in total and LDL-cholesterol levels, especially in women [63,64].

The measurements of systemic oxidative status were part of the nutritional status assessment, in consideration of the relationship between nutrition and haematochemical oxidative stress levels. In fact, several studies suggest that a proper nutritional intake can positively counteract the oxidative and inflammatory state due to stroke insult, improving patients’ neuronal plasticity and recovery [15,24,65]. In our study, on admission, both women and men had elevated oxidative stress levels, according to previous studies from our group [23,32]. However, after the rehabilitation period, men had significantly lower levels of circulating hydroperoxides than women. The cause of these differences remains difficult to determine. It can be hypothesised that reduced oestrogen and progesterone levels in older women may negatively impact the antioxidant protective effect of these hormones, leading to increased oxidative stress [66,67]. At the same time, men may experience higher susceptibility to specific inflammatory conditions that could increase oxidative stress, such as Helicobacter pylori infection and post-stroke gastric ulcers [68]. These factors add further complexity to the interpretation of the findings and require more targeted investigations. These results deserve future studies on a larger sample to confirm whether subacute post-stroke women have a higher vulnerability to oxidative stress and to elucidate the underlying physiological mechanisms.

One interesting result from this study concerns gender differences in food consumption. Given the equal portioning of dishes in our rehabilitation facility, women discarded an average of 23% of “all meals” during hospitalisation, accounting for nearly a quarter of the served dishes. More specifically, women discarded a major quantity of the “first dish” (mainly carbohydrates), the “second dish” (mainly protein), and the “side dish” (mainly vegetables). We can hypothesise that women’s tendency to consume less food than men is influenced by a combination of factors, including metabolic and hormonal ones. Women have a lower resting metabolic rate than men and consequently require lower caloric intake to maintain basic physiological functions [69]. The importance of monitoring the resting metabolic rate in post stroke patients has been recently addressed [70]. However, gender-related metabolic differences have not yet been analysed, despite their importance in preventing malnutrition during hospitalisation. 

Among hormonal factors that can affect food consumption, oestrogen and leptin have important roles, as they regulate weight, food intake, and energy expenditure differently between women and men [26,27,71]. To investigate whether gender differences in food consumption were influenced by nutritional status at admission, we stratified patients into malnourished and non-malnourished groups. Our analysis revealed no gender differences in food consumption within the malnourished group, while a significant difference was present among non-malnourished patients. The absence of gender differences among malnourished patients suggests that factors related to malnutrition itself (e.g., reduced appetite, compromised clinical conditions, weakness) may override gender-specific patterns, leading to a more uniform eating behaviour. In contrast, the presence of gender differences among non-malnourished patients indicates that, in the absence of malnutrition, typical gender-related variations in food consumption become more apparent.

In fact, in addition to gender differences in metabolism and physiological needs, the psychological and cultural approach to food consumption should also be considered. Women may be more susceptible to altering their eating habits in response to emotional states such as stress or depression, which are frequently experienced during hospitalisation and may affect both the quantity and quality of food consumed [72]. Moreover, the more critical approach of Italian women toward meal preparation is an aspect that would merit more attention. It is important to highlight that the lower food intake observed in women, particularly with regard to protein consumption may increase the risk of malnutrition and contribute to the development of sarcopenia, compromising functional recovery during the post-stroke rehabilitation phase.

Women and men in this study also exhibited different trends in rehabilitation outcomes. On admission, women had a similar mBI, while after rehabilitation they reached a significantly lower mBI. Previous studies on subacute post-stroke patients showed that women had more severe symptomatology and worse functional outcomes after rehabilitation [55], together with lower scores in ADL [56,73]. These findings indicate that men are more likely to achieve higher levels of functional independence after rehabilitation than women, suggesting that female gender is an unfavourable prognostic factor in rehabilitation outcomes after stroke. Poggesi et al. [13] analysed 208 post-stroke subjects, among whom women were significantly older than men. Their study showed that, at the time of admission to a rehabilitation hospital, men and women had a similar functional and clinical status and that the functional recovery achieved after inpatient rehabilitation was comparable between the two groups. Furthermore, they observed that women tended to recover better than men in the same age group.

Another important gender disparity found in the present study concerns the MoCA score at admission, adjusted for age and education. This disparity in cognitive impairment seems to diminish after the rehabilitation period. This aspect paves the way for future in-depth investigations to determine whether cognitive differences are concentrated in specific domains. A recent meta-analysis found that when considering cognitive abilities as a whole, gender disparities did not emerge [6]. However, when analysing specific domains, men exhibited lower impairment in attention, executive functions, and language but had a higher risk of verbal memory impairment [6]. Nevertheless, research on gender differences in cognitive impairment among subacute post-stroke patients during rehabilitation remains insufficient. However, this topic deserves more attention due to the association between improved cognitive abilities, particularly executive functions, memory, and visuospatial skills, and increased independence in activities of daily living [74,75,76].

This descriptive study represents a novel contribution to the existing literature. Few studies have examined gender-related differences through a multidimensional assessment. However, this study has some limitations. First, the plate waste does not allow for an accurate calculation of macronutrient, micronutrient, and calorie intake based on the specific needs of each individual. Second, this study is a descriptive analysis conducted on small sample recruited in a single centre. Moreover, we did not consider stroke subtypes, which limits the interpretation of the clinical results. Finally, the lack of precise monitoring of the antioxidant intake represents an additional limitation, underscoring the need for more accurate tracking of supplementation in future studies. Further investigation on a larger sample will allow to overcome these limitations. To this end, we will conduct an analysis of gender differences in a multicentre study involving a large cohort of stroke patients undergoing rehabilitation (trial registered at ClinicalTrials.gov under the identifier NCT06547827).

## 5. Conclusions

From this study, post-stroke women during the rehabilitation period appeared to show higher vulnerability to malnutrition and oxidative stress, combined with lower nutritional intake, highlighting the need for more tailored interventions. These findings suggest the need for early nutritional assessment that is sensitive to gender differences, allowing for the timely identification of patients at higher nutritional or oxidative risk and the development of more personalised dietary and supplementation strategies to improve rehabilitation outcomes. Identifying specific vulnerabilities in women and men offers an innovative perspective that could enhance a “gender-sensitive” rehabilitation approach, promoting more targeted and personalised treatment strategies and optimising recovery in subacute post-stroke patients.

## Figures and Tables

**Table 1:** Baseline characteristics of the whole sample (*n* = 87) and distinguished for women (*n* = 42) and men (*n* = 45). Data are reported as mean ± standard deviation or number and percentage (%). *p* values refer to the Mann–Whitney U-Test with the sample disaggregated by gender.

Baseline Characteristics	Whole Group (*n* = 87)	Women (*n* = 42)	Men (*n* = 45)	*p*-Value
Age (years)	69 ± 12	71 ± 9	66 ± 13	0.110
Index stroke type				
Ischemic	66 (76%)	33 (79%)	33 (73%)	0.568
Haemorrhagic	21 (24%)	9 (21%)	12 (27%)	
Affected side				
Right	43 (49%)	21 (49%)	22 (51%)	0.918
Days from stroke onset to enrolment	105 ± 52	114 ± 57	96 ± 47	0.136
Neglect	16 (18%)	11 (26%)	5 (11%)	0.061
Aphasia	6 (7%)	5 (12%)	1 (2%)	0.129
Smokers and former smokers	45 (54%)	18 (46%)	27 (61%)	0.165
Dysphagia	29 (33%)	16 (38%)	16 (30%)	0.363
Comorbidities				
Hypertension	74 (85%)	35 (83%)	39 (87%)	0.663
Type 2 Diabetes	26 (30%)	11 (26%)	15 (33%)	0.467
Dyslipidaemia	37 (43%)	18 (42%)	19 (42%)	0.952
Prior heart attack	4 (5%)	0 (0%)	4 (9%)	0.048 *
Congestive heart failure	10 (12%)	3 (7%)	7 (16%)	0.219
Atrial fibrillation	12 (14%)	7 (17%)	5 (11%)	0.453
Prior Cancers	8 (9%)	4 (10%)	4 (9%)	0.918
Peripheral vascular disease (PVD)	24 (28%)	11 (26%)	13 (29%)	0.778
Chronic Obstructive Pulmonary Disease (COPD)	10 (12%)	5 (12%)	5 (11%)	0.908
Thyroid Disease	19 (22%)	13 (91%)	6 (13%)	0.047 *
Cumulative Illness Rating Scale (CIRS)				
CIRS severity	2.3 ± 0.4	2.3 ± 0.3	2.3 ± 0.4	0.309
CIRS comorbidity	5.7± 1.7	5.7± 1.7	5.6 ± 1.8	0.570

* *p*-value < 0.05.

**Table 2:** Nutritional status at admission (T0) in terms of anthropometric measurements, screening and diagnosis of malnutrition, nutritional risk index (Geriatric Nutritional Risk Index, GNRI), and haematochemical parameters including oxidative stress panel [hydroperoxides, dROMS; total antioxidants species, BAP; total antioxidants thiol, SHp; oxidative stress index, OSI]. *p* values refer to Mann–Whitney U-test dividing sample by gender. Data are reported as mean ± standard deviation.

Nutritional Status at Admission (T0)	Whole Group (*n* = 87)	Women (*n* = 42)	Men (*n* = 45)	*p*-Value
Anthropometric assessment				
Height (m)	1.66 ± 0.11	1.61 ± 0.07	1.71 ± 0.11	<0.001 ***
Weight (kg)	69.6 ± 17.3	62.8 ± 13.9	75.9 ± 10.8	<0.001 ***
Body Mass Index, BMI (kg/m^2^)	24.9 ± 4.9	24.3 ± 5.4	25.6 ± 4.4	0.058
Nutritional screening				
Mini Nutritional Assessment Short-form (MNA-SF^®^)	7.5 ± 2.3	7.4 ± 2.4	7.7 ± 2.3	0.592
Malnutrition diagnosis (GLIM Criteria)	37 (42.5%)	22 (52.4%)	15 (33.3%)	0.073
Nutritional Risk index				
Geriatric Nutritional Risk Index (GNRI)	105.2 ± 12.8	103.8 ± 14.2	106.5 ± 11.4	0.248
Haematochemical parameters				
Albumin (g/dL)	3.8 ± 0.5	3.7 ± 0.5	3.9 ± 0.5	0.332
Glucose (mg/dL)	107.1 ± 37.6	113.1 ± 43.4	101.6 ±30.7	0.315
Total Cholesterol (mg/dL)	123.6 ± 37.6	131.6 ± 41.4	116.2 ± 32.4	0.198
HDL cholesterol (mg/dL)	54.5 ± 17.0	58.6 ± 14.3	50.7 ± 18.5	0.006 **
Ratio (Total cholesterol/HDL cholesterol)	2.4 ± 0.9	2.3 ± 0.8	2.5 ± 0.9	0.545
Triglycerides (mg/dL)	124.0 ± 62.4	120.3 ± 65.2	127.5 ± 60.2	0.415
Calcium (mg/dL)	8.8 ± 1.1	8.9 ± 1.2	8.8 ± 1.0	0.424
Magnesium (mg/dL)	1.8 ± 0.3	1.8 ± 0.3	1.7 ± 0.3	0.068
Iron (µg/dL)	64.2 ± 19.7	67.3 ± 16.8	61.8 ± 21.8	0.263
Oxidative stress panel				
dROMs (UCARR)	544 ± 125	556 ± 145	532 ± 102	0.764
BAP (µmol/L)	2284 ± 458	2301 ± 512	2268 ± 406	0.796
SHp (µmol/L)	626 ± 125	693 ± 141	613 ± 106	0.643
Oxidative stress index (OSI = BAP/dROMs)	4.4 ± 1.3	4.4 ± 1.5	4.4 ± 1.2	0.773

** *p*-value < 0.01; *** *p*-value < 0.001.

**Table 3:** Nutritional status after six weeks of rehabilitation (T1) in terms of anthropometric measurements, nutritional risk index (Geriatric Nutritional Risk Index, GNRI), and haematochemical parameters including oxidative stress panel [hydroperoxides, dROMS; total antioxidants species, BAP; total antioxidants thiol, SHp; oxidative stress index, OSI]. *p* values refer to Mann-Whitney U-test dividing sample by gender. Data are reported as mean ± standard deviation.

Nutritional Status After Rehabilitation (T1)	Whole Group (*n* = 87)	Women (*n* = 42)	Men (*n* = 45)	*p*-Value
Anthropometric assessment				
Weight (kg)	69.2 ± 16.8	62.5 ± 13.5	75.5 ± 17.3	<0.001 ***
Body Mass Index (BMI) (kg/m^2^)	24.8 ± 4.7	24.2 ± 5.2	25.4 ± 4.1	0.080
Nutritional risk index				
Geriatric Nutritional Risk Index (GNRI)	107.6 ± 12.7	107.9 ± 14.9	107.4 ± 10.6	0.889
Haematochemical parameters				
Albumin (g/dL)	4.0 ± 0.5	4.0 ± 0.5	4.0 ± 0.4	0.871
Glucose (mg/dL)	103.3 ± 41.7	102.2 ± 41.4	104.3 ± 42.3	0.959
Total Cholesterol (mg/dL)	127.9± 62.4	143.0 ±53.2	113.9 ± 37.8	0.013 *
HDL cholesterol (mg/dL)	54.6 ± 16.1	61.5 ± 17.1	48.1 ± 12.1	<0.001 ***
Ratio (Total cholesterol/HDL cholesterol)	2.5 ± 0.9	2.5 ± 1.1	2.5 ± 0.8	0.586
Triglycerides (mg/dL)	121.9 ± 59.0	129.4 ± 50.9	114.9 ±65.5	0.051
Calcium (mg/dL)	8.8 ± 1.0	9.0 ± 1.1	8.7 ± 0.9	0.062
Magnesium (mg/dL)	1.7 ± 0.4	1.7 ± 0.3	1.6 ± 0.4	0.685
Iron (µg/dL)	60.9 ± 18.5	63.0 ± 22.0	59.1 ± 14.8	0.671
Oxidative stress panel				
dROMs (UCARR)	520 ± 135	549 ± 143	491 ± 121	0.041 *
BAP (µmol/L)	2345 ± 479	2364 ± 515	2328 ± 448	0.797
SHp (µmol/L)	640 ± 126	643 ± 132	637 ± 120	1.000
Oxidative stress index (OSI = BAP/dROMs)	4.8 ± 1.5	4.6 ± 1.5	4.6 ± 1.5	0.154

* *p*-value < 0.05; *** *p*-value < 0.001.

## Data Availability

The data supporting the findings of this study are available from the corresponding author upon reasonable request.

## Supplementary Materials

The following supporting information can be downloaded at https://www.mdpi.com/article/10.3390/neurolint17120193/s1, Table S1: The checklist completed in accordance with the Strengthening the Reporting of Observational studies in Epidemiology (STROBE 2019) guidelines; Figure S1: Flow chart of the observational study.
