# Supplementary figures and images for: Gender Differences in Subacute Post-Stroke Patients During Rehabilitation: Functional, Cognitive, and Nutritional Insights
Source: Neurol Int. 2025 Nov 30;17(12):193. doi: 10.3390/neurolint17120193 (PMC12735716; doi:10.3390/neurolint17120193)

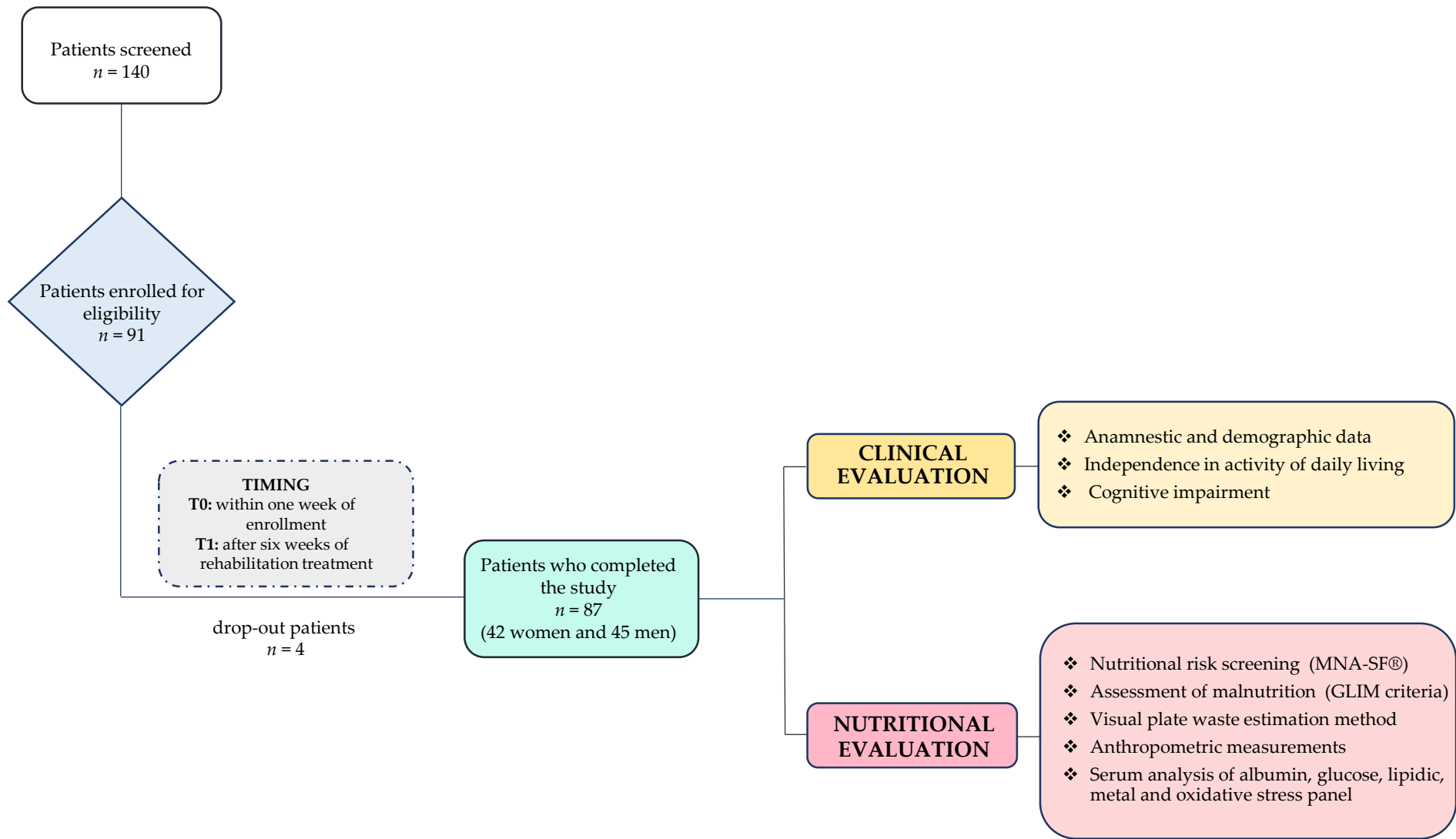

Supplement: Supplementary file 1 [file neurolint-17-00193-s001.zip › Figure S1-FlowChart_Cocco.pdf]
